# Smaller bladder capacity and stronger bladder contractility in patients with ketamine cystitis are associated with elevated TRPV1 and TRPV4

**DOI:** 10.1038/s41598-021-84734-4

**Published:** 2021-03-04

**Authors:** Hsueh-Hui Yang, Jia-Fong Jhang, Yung-Hsiang Hsu, Yuan-Hong Jiang, Wei-Jun Zhai, Hann-Chorng Kuo

**Affiliations:** 1 Department of Medical Research, Hualien Tzu Chi Hospital, Buddhist Tzu Chi Medical Foundation, Hualien, 970 Taiwan; 2 Department of Urology, Hualien Tzu Chi Hospital, Buddhist Tzu Chi Medical Foundation and Tzu Chi University, 707, Sec. 3, Chung Yang Rd., Hualien, 970 Taiwan; 3 Department of Pathology, Hualien Tzu Chi Hospital, Buddhist Tzu Chi Medical Foundation and Tzu Chi University, Hualien, 970 Taiwan

**Keywords:** Urology, Bladder

## Abstract

Stronger contractility and smaller bladder capacity are common symptoms in ketamine cystitis (KC). This study investigates the association between expression levels of transient receptor potential cation channel subfamily V (TRPV) proteins and the clinical characteristics of KC. Bladder tissues were obtained from 24 patients with KC and four asymptomatic control subjects. Video urodynamic parameters were obtained before surgical procedures. The TRPV proteins were investigated by immunoblotting, immunofluorescence staining, and immunohistochemistry. The Pearson test was used to associate the expression levels of TRPV proteins with clinical characteristics of KC. The expression level of TRPV1 and TRPV4 was significantly higher in the severe KC bladders than in mild KC or control bladders. The TRPV1 proteins were localized in all urothelial cell layers, and TRPV4 was located in the basal cells and lamina propria. The expression of TRPV1 was negatively associated with maximal bladder capacity (r = − 0.66, *P* = 0.01). The expression of TRPV4 was positively associated with the velocity of detrusor pressure rise to the maximum flow rate (r = 0.53, *P* = 0.01). These observations suggest smaller bladder capacity and stronger contractility in KC are associated with an elevated expression of TRPV1 and TRPV4, respectively.

## Introduction

Ketamine can inhibit the function of the N-methyl-d-aspartate receptor and is a commonly used anesthetic agent in human and veterinary procedures. In recent years, its easy accessibility, low price, and relatively short-acting effect have led to an increase in its illegal use by teenagers as a recreational drug. It is categorized as a schedule 3 controlled drug in Taiwan and has emerged as the most common illegal drug^1^. In 2007, Shahani et al. reported a new clinical entity of ulcerative cystitis related to chronic ketamine use^2^. It is a florid nonbacterial cystitis condition called ketamine cystitis (KC) ^2,3^. These patients usually experience urgency, severe bladder pain, and small bladder capacity.

The precise cause of KC is still not clear, although several possible pathologic mechanisms have been proposed. One of the mechanisms suggests that direct toxicity by ketamine or its metabolites on the urothelial cells causes an inflammatory response, inducing interstitial fibrosis and disrupting the proliferation, or activating the intrinsic apoptotic pathway^3–6^. Enhanced oxidative stress is another potential mechanism that may contribute to urothelial barrier defects and bladder cell apoptosis^7^. Although downregulated urothelial structural proteins have been observed in KC patients^8,9^, intact urothelial barrier function and morphology have been reported in KC mice, which indicates that disrupted urothelial barrier function may not be the direct cause of KC^10^. A recently demonstrated novel pathway that results in ketamine-induced smooth muscle dysfunction happens through the inhibition of the L-type voltage-gated calcium channel^11^.

Urothelium is more than a barrier protecting the bladder stroma and signaling the bladder’s voiding function^12^. It acts as a sensory structure to control the bladder’s contractile activity, which is one of the parameters used to evaluate the voiding function of the bladder. Recently, receptors in the urothelium such as the P2X, M2, M3, p75 low-affinity nerve growth factor receptor, and β-3 adrenergic receptors have been shown to participate in the pathophysiology of KC^9,13,14^.

The other possible sensor molecules in the urothelium are ion channels. Transient receptor potential (TRP) channels expressed in the lower urinary tract are thought to be involved in the bladder’s pathologic function^15^. However, to our knowledge, little research has been done to study what roles these proteins have in KC. Only one research group used TRP cation channel subfamily V member 1 (TRPV1) as a marker to evaluate the effect of lipotoxin and Ba‐Wei‐Die‐Huang‐Wan treatment on the KC rat model^16,17^. Instead of using an animal model, the present study used human patients’ bladder samples to investigate the associations between the expression levels of TRPV proteins and the clinical characteristics of KC. The results of this study may reveal the possible roles of these proteins in KC.

## Results

### Clinical data

A total of 24 patients with KC (17 men and seven women, mean age 27.8 ± 5.1 years) and four control subjects (two men and two women, mean age 55.0 ± 4.2 years) were recruited. Table 1 lists the profile, urodynamic parameters, and Western blot analysis results of TRPV1 and TRPV4 levels for the different patient groups. Among the patients with KC, 12 were classified as mild KC (10 men and two women, mean age 28.2 ± 4.9 years), and 12 were classified as severe KC (seven men and five women, mean age 27.4 ± 5.2 years).

**Table 1:** Clinical characteristics and Western blot analysis data of KC patients and control subjects.

	Control	KC			*P* value^a^	*P* value^b^
		Total	Mild KC	Severe KC		
Number	4	24	12	12		
Gender	M2, F2	M17, F7	M10, F2	M7, F5		
Age (years)	55.0 ± 4.2	27.8 ± 5.1	28.2 ± 4.9	27.4 ± 5.2	< 0.001	0.718
**VUDS**						
CBC (ml)	NA	71.3 ± 54.9	138.8 ± 50.5	48.9 ± 12.2		0.001
MBC (ml)	NA	215.7 ± 128.9	333.3 ± 68.3	127.5 ± 82.2		0.003
ΔBC (ml)	NA	137.0 ± 86.1	194.5 ± 60.6	87.7 ± 75.0		0.022
P_det_ (cmH_2_O)	NA	34.3 ± 26.6	30.6 ± 13.1	38.3 ± 36.9		0.743
Q_max_ (ml/s)	NA	8.8 ± 5.5	10.7 ± 6.1	6.3 ± 3.7		0.125
V_Pdet.max_ (cmH_2_O/s)	NA	4.2 ± 2.4	3.1 ± 1.1	5.3 ± 2.9		0.076
VAS	NA	6.4 ± 2.8	2.2 ± 1.5	7.5 ± 2.3		0.002
**Western blot analysis**						
TRPV1/GAPDH	0.08 ± 0.04	0.58 ± 0.54	0.25 ± 0.27	0.91 ± 0.55	0.017	0.002
TRPV4/GAPDH	0.02 ± 0.01	0.27 ± 0.28	0.08 ± 0.04	0.47 ± 0.29	0.002	< 0.001

The Western blot analysis was normalized against GAPDH.

*CBC* cystometric bladder capacity, *MBC* maximal bladder capacity, *ΔBC* the difference between MBC and CBC, *P*_*det*_ detrusor pressure, *V*_*Pdet.max*_ velocity to reach maximal P_det_ (cm H_2_O/s, defined as the slope of pressure to time from the start of detrusor contraction to reach P_det.max_), *Q*_*max*_ maximum flow rate, *VAS* visual analog score.

^a^*P* values between all KC patients and controls, ^b^*P* values between mild KC and severe KC patients.

**Figure 1:**
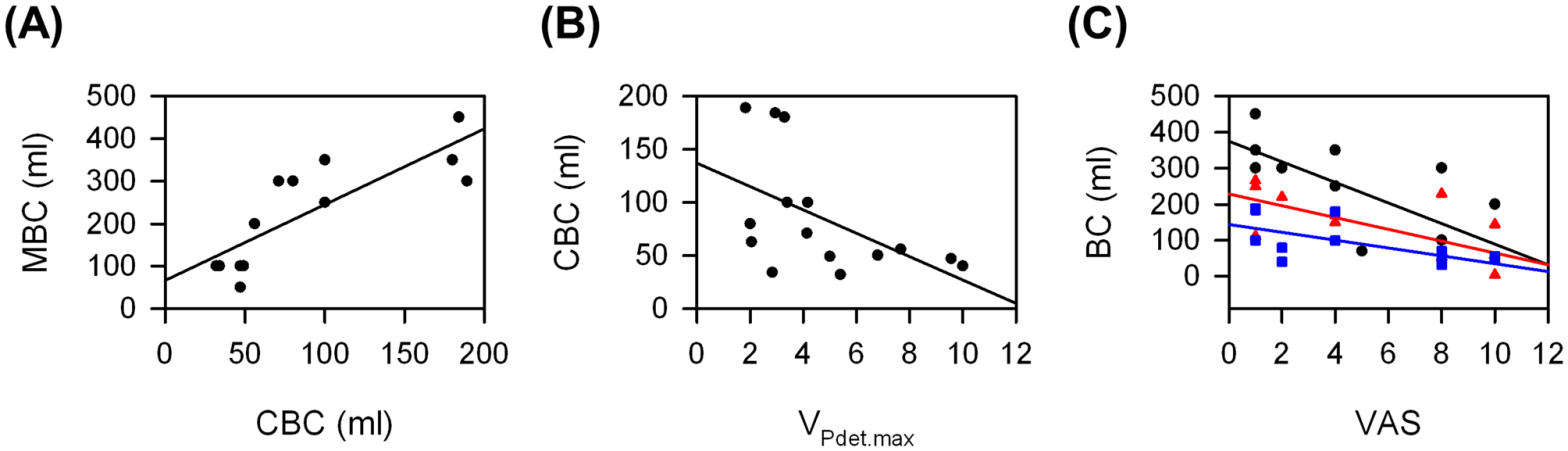
Significant associations between urodynamic parameters from KC patients: (**A**) between MBC and CBC, (**B**) between CBC and V_Pdet.max_, and (**C**) between MBC (black circle), ΔBC (red triangle), or CBC (blue square) and VAS.

Both the maximal bladder capacity (MBC) and cystometric bladder capacity (CBC) were significantly smaller in patients with severe KC than in those with mild KC. On the other hand, the visual analogue scale (VAS) of pain and velocity to reach maximal detrusor pressure (V_Pdet.max_) were significantly greater in bladders with severe KC than in those with mild KC. Figure 1 shows the associations between these parameters. There was a significantly positive association between MBC and CBC (r = 0.82, *P* < 0.001). The MBC, CBC, and the difference between MBC and CBC (ΔBC) were significantly negatively associated with VAS (r = − 0.75, *P* = 0.002; r = − 0.68, *P* = 0.006; and r = − 0.67, *P* = 0.01, respectively). Only CBC was significantly negatively associated with V_Pdet.max_ (r = − 0.53, *P* = 0.04).

### Elevated TRPV1 and TRPV4 in patients with KC

**Figure 2:**
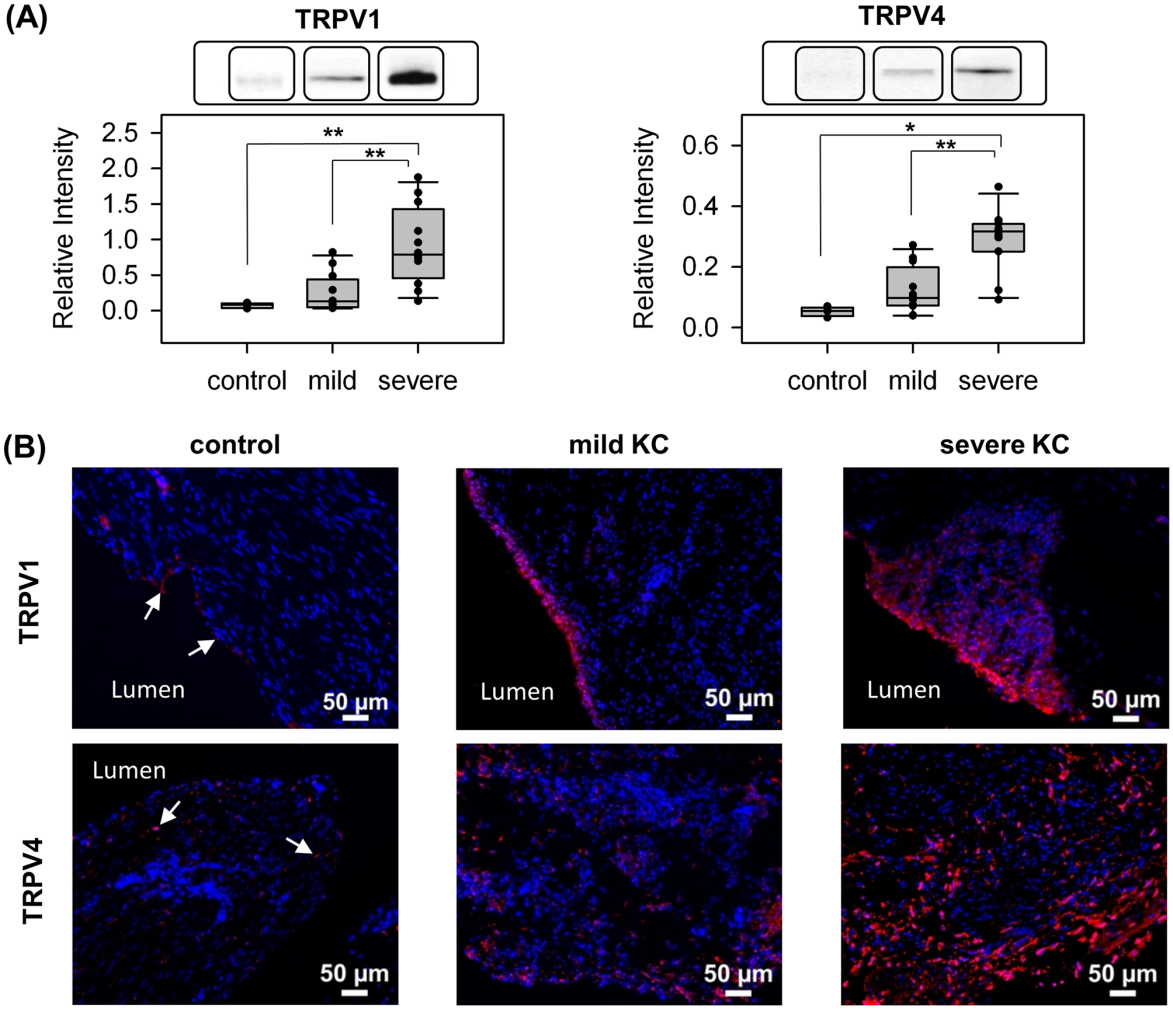
Higher expression of TRPV1 and TRPV4 in KC bladders than in control bladders. (**A**) Representative results of Western blot analysis (from different experiments) and scatter plot of the quantification of TRPV1 and TRPV4 to GAPDH in control, mild KC, and severe KC bladder specimens. The bar graph was quantified using ImageMaster TotalLab. *P < 0.05 and **P < 0.01. Images for GAPDH and from all samples are available in Supplementary Fig. S1. (**B**) Representative results of immunofluorescence staining with TRPV1 and TRPV4 in control (arrows), mild KC, and severe KC bladder specimens. Stronger TRPV1 and TRPV4 fluorescence (red) was detected in severe KC specimens than in mild KC specimens. Nuclei were labeled with DAPI (blue).

Western blot analysis was performed to assay the protein expression level in bladder specimens of all 24 patients with KC and of the four control subjects. Figure 2A and Table 1 show the results of Western blot analysis. Patients with KC had a significantly higher expression of TRPV1 and TRPV4 than control subjects. The expressions of TRPV1 and TRPV4 in the bladders of patients with severe KC were significantly higher than in the bladders of those with mild KC.

Immunostaining was subsequently used to validate the results of Western blot analysis. However, because the urothelium is prone to nonspecific adsorption of antibodies, a competitive binding assay using a blocking peptide was first performed to determine the antibody’s binding specificity. Only the TRPV4 antibody was tested because the blocking peptide (101 amino acids) of TRPV1 was not available. The TRPV4 staining disappeared when the antibody was preincubated with its blocking peptide, indicating the stain was specific (Supplementary Fig. S2). The immunofluorescence staining from similar bladder regions shown in Fig. 2B indicated that the protein expressions of TRPV1 and TRPV4 in the bladders of control subjects were lower than those of KC patients (semi-quantification was in the Supplementary Table S1), which is consistent with the Western blot analysis.

### Localization of TRPV1 and TRPV4 in the bladder

**Figure 3:**
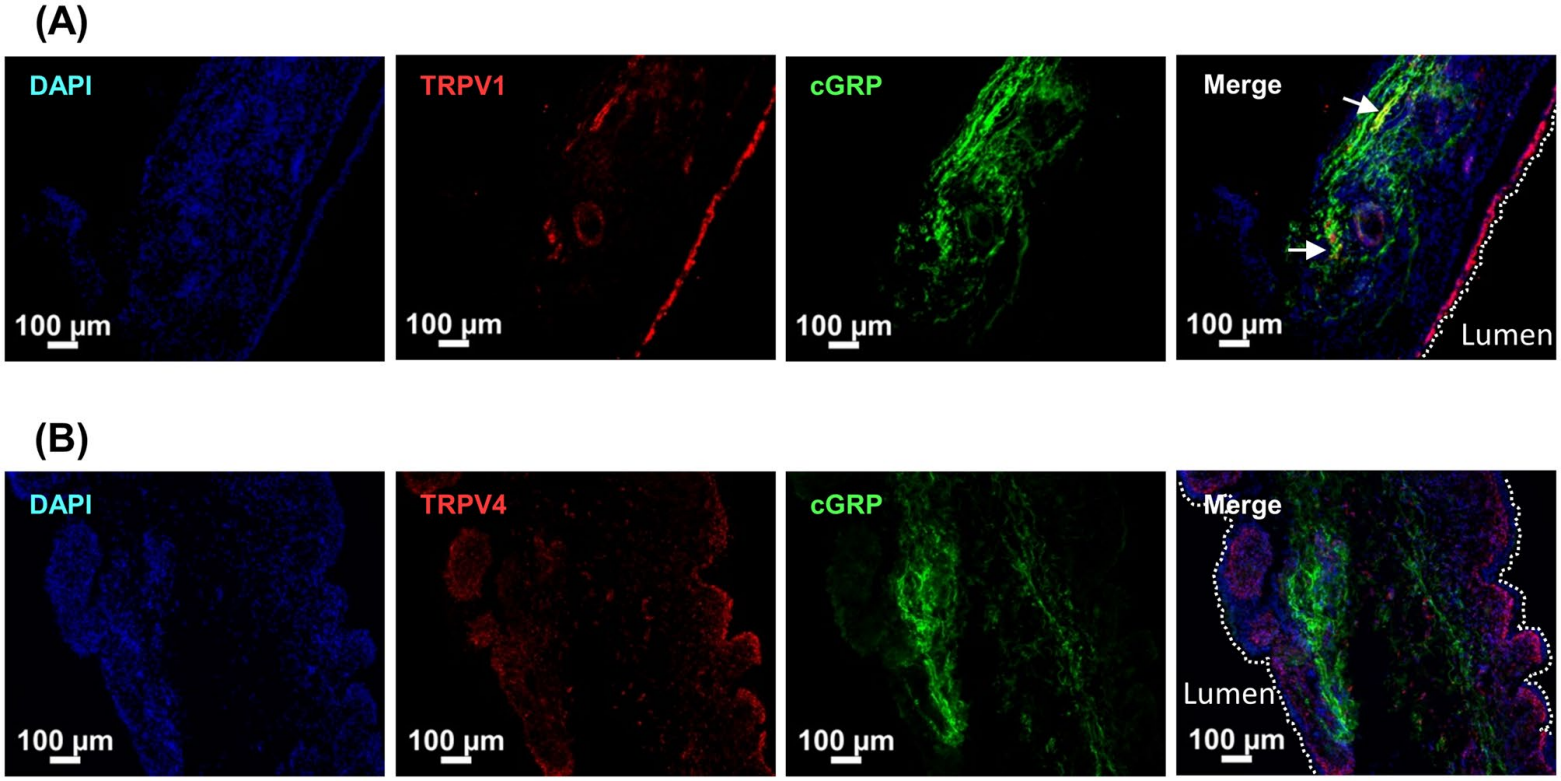
Representative results of immunofluorescence localization of TRPV1 (**A**) and TRPV4 (**B**) in mild KC bladder specimens. Double immunofluorescence of TRPV1 or TRPV4 (red) with CGRP (green) was performed. Nuclei were labeled with DAPI (blue). TRPV1-positive nerve fibers are indicated by arrows. Dashed white lines indicate the apical surface of the umbrella cells. The double immunochemical staining revealed that the lack of co-expression with CGRP for TRPV4, and the expression of TRPV1 in only a few nerve fibers.

**Figure 4:**
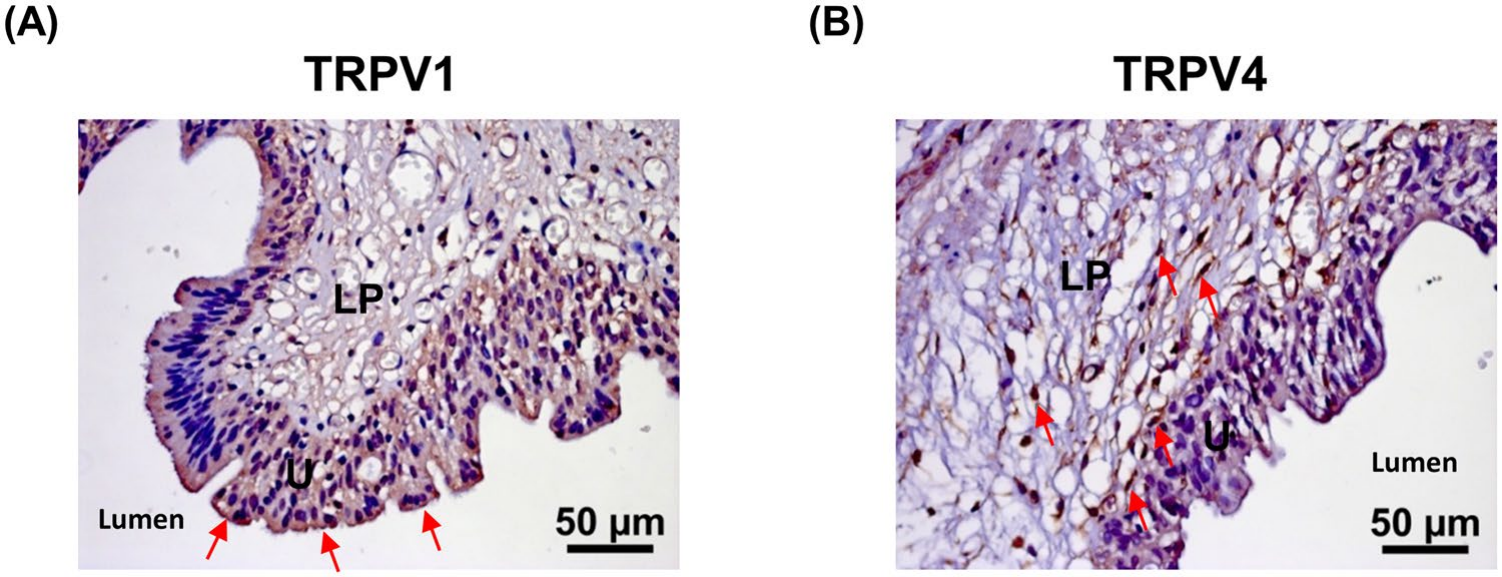
Representative results of IHC of TRPV1 (**A**) and TRPV4 (**B**) in severe KC bladder specimens. Arrows indicate TRPV1 or TRPV4 positive cells. The expression of TRPV1 is mainly distributed in all urothelial cell layers. In contrast, TRPV4 was found in the basal cells and lamina propria. *U* urothelium, *LP* lamina propria.

Calcitonin gene-related peptide (CGRP), a neuron-specific marker, typically presents in the nerves distributed within the suburothelium region^18^. Both TRPV1 and TRPV4 were subsequently costained with CGRP to study the localization of these proteins. The results of immunofluorescence double-staining for TRPV4 and TRPV1 with CGRP are shown in Fig. 3. Only a few TRPV1-positive nerve fibers (yellow) were found. No TRPV4-positive nerve fibers were found. To further illustrate the localization of TRPV1 and TRPV4, Immunohistochemistry (IHC) was then carried out. Figure 4 shows that TRPV1 was distributed in all urothelial cell layers. On the other hand, TRPV4 was found in the basal cells and lamina propria.

### Association between urodynamic parameters and expressions of TRPV1 and TRPV4

**Figure 5:**
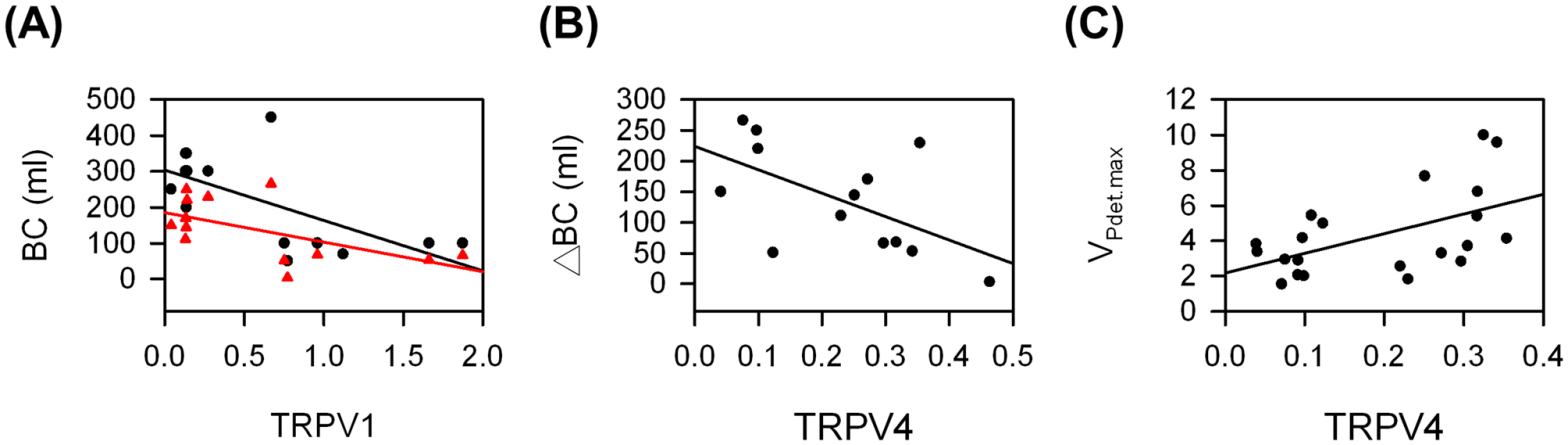
Significant associations between urodynamic parameters and the expression of TRPV1 or TRPV4 form KC patients: (**A**) between MBC (black circle) or ΔBC (red triangle) and TRPV1, (**B**) between ΔBC and TRPV4, and (**C**) between V_Pdet.max_ and TRPV4.

**Figure 6:**
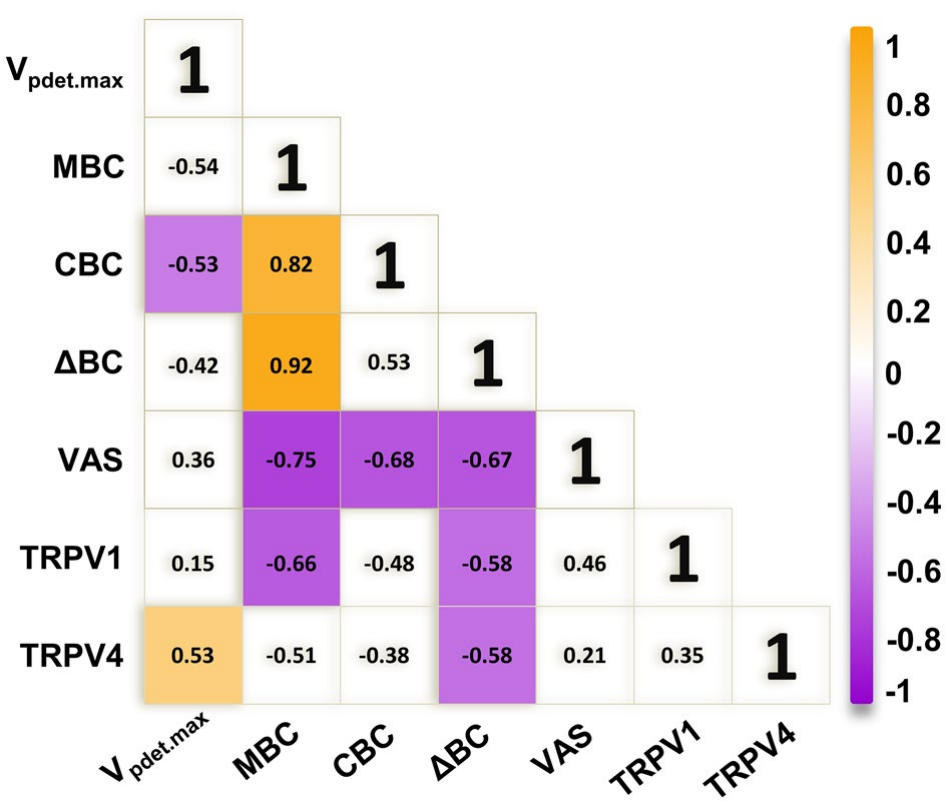
Summary of association between urodynamic parameters and TRPVs. Colored boxes represent a significant association coefficient between the variables (*P* < 0.05). Chrome yellow and purple indicate positive and negative associations, respectively.

To study the possible roles of TPRV1 and TRPV4 in KC, the association between TRPV1 and TRPV4 and between these two proteins and urodynamic parameters were evaluated. Figure 5 shows the scatter plots with significant association and all the association results were summarized in Fig. 6. Increased expressions of both TRPV1 and TRPV4 were observed in the bladders of KC patients, but they were not significantly associated (r = 0.35, *P* = 0.09). In addition, the expression of TRPV1 was significantly negatively associated with MBC and ΔBC (r = − 0.66, *P* = 0.01 and r = − 0.58, *P* = 0.04, respectively). However, the expression of TRPV4 was significantly positively associated with V_Pdet.max_ (r = 0.53, *P* = 0.01) and significantly negatively associated with ΔBC (r = − 0.58, *P* = 0.04).

## Discussion

Although there have been studies of TRPV1 and TRPV4 in the bladders, most were based on animal tissue or cell culture models. Only a few studies were conducted on human bladders. The data presented in the current study are from bladder tissue of patients with mild and severe KC. The data show, for the first time, that expression levels of TRPV1 and TRPV4 observed in patients with severe KC are significantly increased over those in patients with mild KC and in control subjects. According to our previous report, KC patients usually show significantly greater V_Pdet.max_ and smaller bladder capacity than control subjects^19^. In the present study, the association between different urodynamic parameters and between urodynamic data and protein expression were studied. The results summarized in Fig. 6 reveal a significant association between (1) reduced bladder capacity and increased VAS of pain, (2) increased TRPV1 expression and reduced bladder capacity, and (3) increased TRPV4 expression and increased V_Pdet.max_.

Both TRPV1 and TRPV4 are nonselective cationic channels activated by a diversity of stimuli such as heat, acidity, and chemicals. Because mechanical and chemical stimuli also stimulate bladder functions, these two proteins are likely urothelial sensors for bladder distention. Thus, they are interesting targets for the study of controlling sensory and motor activity of the bladder. However, although many investigations have been done on the expression, distribution, and functions of TRPV1 and TRPV4 in the bladder, the conclusions of the role of TRPV1 and TRPV4 in bladders are still unclear.

The expression of TRPV1 was originally reported to be exclusively expressed in sensory ganglia^20^. Birder et al. were the first one to describe the presence of TRPV1 in the urothelial cells from rats^21^. Although subsequent studies have not been able to reproduce the expression of TRPV1 in mouse urothelial cells, the presence of nerve terminals that extend into the urothelium has been reported^22,23^.

Similarly, the localization of TRPV1 in human bladders is also controversial. The non-neuronal localization of TRPV1 in detrusor smooth muscle cells and interstitial cells of Cajal and the localization of TRPV1 in urothelial cells have been reported^24–27^. In accordance with the previous reports^26,27^, the expression of TRPV1 was observed in both the urothelium and suburothelium in the present study. The urothelium layerwas labeled stronger than suburothelium layers. As the severity of KC increased, TRPV1 was distributed into deeper bladder layers. However, only a few TRPV1 stains of the suburothelium were positively confirmed as neuronal processes by co-staining with CGRP.

Several studies have shown higher TRPV1 expression in patients with overactive bladder^26–29^. When patients with neurogenic detrusor overactivity (DO) responded to intravesical resiniferatoxin (RTX) therapy, a significant decrease in TRPV1-immunoreactive nerve fibers was observed^26^. This significant decrease in TRPV1-immunoreactivity was only noted in the basal cell layer, and the percentage of changes was comparable to the changes in suburothelial TRPV1 nerve fiber density^27^. However, for idiopathic DO patients with successful intravesical RTX treatment, TRPV1 was observed to be overexpressed in the urothelium and suburothelium^28^. Despite different localizations of TRPV1 in these studies, the involvement of TRPV1 in the pathophysiology of DO was suggested. In this study, the expression of TRPV1 was significantly greater in both the urothelium and suburothelium of specimens from patients with severe KC compared with those from patients with mild KC or control subjects. This suggested that TRPV1 might also be involved in the pathophysiology of KC.

In our study, a significant negative association between the expression of TRPV1 with MBC was observed. The possible role of TRPV1 in bladder function has been studied using TRPV1-knockout animals. Daly et al. reported attenuated low threshold afferent responses in TRPV1-knockout mice but an unchanged high-threshold afferent sensitivity, suggesting that neuronal TRPV1 channels in the suburothelium are needed in the excitability of low-threshold bladder afferents^30^. Birder et al. demonstrated an increase in bladder capacity in anesthetized TRPV1-knockout mice but an unaffected micturition frequency in conscious TRPV1-knockout mice. This suggests that TRPV1-mediated mechanisms are responsible for setting the micturition threshold under anesthesia^31,32^. These studies revealed the negative association between bladder capacity and the expression of TRPV1, which is consistent with our observation.

With regard to TRPV4, it is a broadly expressed ion channel in the body, such as the central and peripheral nervous system, hair, skin, and the bladder. Within the rat and mouse urinary bladder, the localization of TRPV4 has been demonstrated mainly in the plasma membrane of the intermediate and basal cells and to a less extent in the detrusor smooth muscle cells^22,23,32–35^. Our observations agree with these reports in that we observed that TRPV4 is localized in the basal cells and lamina propria.

Apart from the localization of TRPV4 in the bladder, our results also revealed that the expression of TRPV4 increased as the severity of KC increased, indicating that the possible involvement of TRPV4 in the pathophysiology of KC could not be ruled out. Similarly, the increased expression of TRPV4 was detected in the bladder urothelium in rats subjected to repeated variate stress (RVS)^34^. Studies indicated that the intravesical administration of the TRPV4 antagonist HC067047 to block TRPV4 could ameliorate decreased bladder capacity and increased voiding frequency in both RVS and cyclophosphamide-induced cystitis animals^34,36^. More recently, the TRPV4 agonist, GSK1016790A, was used to treat detrusor underactivity. After the intravesical application of GSK1016790A, increased voiding frequency and reduced bladder capacity, voided volume, and post-void residuals were observed, without an increase in nonvoiding contractions^35,37^. Based on these studies, our observation of increased expression of TRPV4 indicates that TRPV4 might participate in the KC symptoms of decreased bladder capacity and increased voiding frequency.

Previously, Gevaert et al. made two observations. First, the TRPV4-knockout mice exhibited a lower frequency of voiding contractions and a higher frequency of nonvoiding contractions. Second, the amplitude of spontaneous contractions in explanted bladder strips and intravesical stretch-evoked ATP release in isolated whole bladders from TRPV4-knockout mice were significantly reduced^32^. Based on these observations, they raised the possibility that TRPV4 plays a critical role in urothelium-mediated transduction of intravesical mechanical pressure. To confirm this possibility, Mochizuki et al. established a cell-stretch system to investigate stretch-evoked changes in intracellular Ca^2+^ concentration and ATP release, and further indicated that TRPV4 induces robust Ca^2+^ influx and contributes to ATP release upon extension. Thus TRPV4 is critically involved in the sensing mechanical stretch stimuli in the bladder^33^. On the other hand, Janssen et al. suggested that TRPV4 channels could be activated by urothelial stretch because TRPV4 channels are connected to adherence junctions and the actin cytoskeleton^38,39^. Taken together, the significantly positive association between the expression of TRPV4 with V_Pdet.max_ in the present study might be correlated with the mechanosensory properties of TRPV4.

To our knowledge, this is the first study that uses human samples to provide the association between urodynamic parameters and the expression of TRPV1 and TRPV4. Although the present study demonstrated remarkable changes in the expression of TRPV1 and TRPV4 and a significant association between the expression of these proteins and the clinical characteristics of KC, there are still limitations. First, only a small number of patients was studied and a larger sample size should be investigated in the future. Second, control subjects were not age-matched with KC patients, and there were no urodynamic parameter data for control subjects. Therefore, the association between urodynamic parameters and the protein expression can only be obtained between patients with mild and severe KC and not between KC patients and control subjects. Third, the tissue collection was not the same in all analysed groups resulting only bladder tissue form severe KC contained muscle layers. However, this problem could be solved using immunostaining from the similar region of the bladders.

In conclusion, this study reveals that in KC patients, a higher degree of VAS is associated with a smaller value of MBC and CBC, and a greater magnitude of V_Pdet.max_ is associated with a smaller value of CBC. Our data also show that both TRPV1 and TRPV4 are upregulated in the urothelium of KC patients, and the degree of upregulation increases with the degree of severity. Elevated TRPV1 and TRPV4 are associated with the smaller MBC and the greater V_Pdet.max_, respectively. Detailed studies are needed to prove the possible involvements of these two channels as mechanosensors in the pathogenesis of KC bladders.

## Materials and methods

### Patients

Twenty-four patients with proven KC and four control subjects were enrolled in the study. The control subjects were patients with bladder cancer or prostate cancer undergoing radical surgery without urinary tract infection or irritative bladder symptoms. Of the patients with proven KC, 12 showed signs of mild bladder dysfunction (defined as MBC ≥ 300 ml under cystoscopic hydrodistention) and 12 showed signs of severe bladder dysfunction (defined as MBC < 300 ml under cystoscopic hydrodistention). The inclusion criteria for KC included the regular misuse of ketamine for more than six months and new onset of lower urinary tract symptoms without bacterial urinary tract infection, stone disease, or malignancy. Computed tomography and cystoscopic hydrodistention confirmed that the patients with KC had a contracted bladder with severe erosive bladder mucosa and profuse bleeding after hydrodistention.

### Video urodynamic study

Patient evaluations included history taking, physical examination, clinical symptoms, VAS of pain, video urodynamic study (VUDS), cystoscopy, and renal ultrasound. During VUDS, a 6-Fr transurethral dual-channel catheter was inserted into the urinary bladder to record the intravesical pressure (P_ves_) and post-void residual urine volume. The intra-abdominal pressure (P_abd_) was measured by placing an 8-Fr catheter mounted with a water-filled balloon. Perineal surface electrodes were placed for external sphincter electromyography. The VUDS was routinely performed at least twice to confirm bladder and bladder outlet conditions during storage and voiding phases by infusing 20% urography in saline at a rate of 10–20 ml/min. The detrusor pressure (P_det_) was calculated by subtracting the P_abd_ from the P_ves_ electronically. A C-arm cinefluoroscope was used to visualize the bladder neck and urethra during the filling and voiding phases. The urodynamic parameters were recorded, including P_ves_, P_det_, CBC, and maximum flow rate (Q_max_). During the voiding phase, the slope of detrusor pressure rise to reach Q_max_ was calculated as V_Pdet.max_. The terminology used in this study followed the recommendations of the International Continence Society^40^.

### Cystoscopic hydrodistention and bladder tissue retrieval

All patients underwent cystoscopic hydrodistention under anesthesia at an intravesical pressure of 80 cm of water, and bladder glomerulations and MBC were recorded. The difference between MBC and CBC was defined as ΔBC, indicating the residual detrusor distensibility under anesthesia. Random biopsies of the posterior bladder wall were obtained after cystoscopic hydrodistention. Each specimen was 2 mm in diameter, and only bladder mucosa was obtained. The bladder biopsy specimens were sent to the hospital pathology department for investigation of malignancy.

Patients were treated conservatively by intravesical hyaluronic acid instillations, non-steroid anti-inflammatory drugs, or botulinum toxin A injections. Patients who continued to have bladder pain, severe urinary frequency, and a small MBC less than 150 ml after cystoscopic hydrodistention and conservative management were offered augmentation enterocystoplasty with partial cystectomy for rapid relief of symptoms and early return to work^41^. Bladder tissue was harvested from the partial cystectomy specimen.

The study was approved by the institutional review board and Ethics Committee of the Buddhist Tzu Chi General Hospital (IRB number 104-163-A). The bladder tissue samples were collected after obtaining an informed consent form. All research activities were performed in accordance with the guidelines and regulations of the Declaration of Helsinki. Bladder specimens were retrieved from partial cystectomy in patients with severe KC and from bladder biopsies in those with mild KC. The bladder biopsies were taken at the same sites in the control subjects and prepared using the same methods.

### Protein extraction

Bladder tissue was dissected out and homogenized in liquid nitrogen. The bladder powder was then transferred to centrifuge tubes containing lysis buffer and centrifuged at 4 °C for 20 min at 16,000 g. The remaining insoluble pellet was discarded and the soluble bladder protein extracts were either used immediately or stored at − 80 °C. The protein concentration was measured using a Bio-Rad protein assay kit.

### Western blot analysis

Western blot analysis was performed similarly to previously described^19^. Tissue lysates from 12 patients with mild KC, 12 patients with severe KC, and four control subjects were separated by 1-dimensional sodium dodecyl sulfate–polyacrylamide gel electrophoresis and transferred onto an Immobilon-P polyvinylidene fluoride transfer membrane (Millipore, Bedford, MA) by electroblotting. The first applied antibodies included anti-TRPV1 and anti-TRPV4 (Abcam, #ab111973 and #ab94868, Cambridge, MA). The second applied antibody was IgG-conjugated horseradish peroxidase (anti-rabbit, Gene Tex, Inc., Irvine, CA). Signals were visualized by the enhanced chemiluminescence kit (BioRad, Madrid, Spain). The scanned films were quantified using ImageMaster TotalLab, Version 2.01 (GE Healthcare, Piscataway, NJ), and data were expressed as relative fold to GAPDH.

### Immunofluorescence staining

Immunofluorescence stain was performed according to the previous method^42^. The urinary bladder specimens were first fixed with an ice-cold solution of 4% formaldehyde in phosphate-buffered saline (PBS, pH 7.4) and then rinsed with ice-cold PBS containing 15% sucrose. Four sections per specimen were cut using a cryostat at a thickness of 8 μm and collected on new silane III-coated glass slides. The sections were then incubated overnight at 4 °C with primary antibodies (-CGRP, Abcam, #ab81887, Cambridge, MA, and others were the same as used in Western blotting), rinsed with 0.1% Tween-20 in PBS, and incubated with conjugated Alexa 594 secondary antibodies (Thermo Fisher Scientific, Waltham, MA). The sections were counterstained with 4′,6-diamidino-2-phenylindole (Sigma Chemical Company, St. Louis, MO). Negative controls included the isotype of the primary antibody.

### Peptide competition assay for TRPV4 antibody

The TRPV4-blocking peptide corresponding to the amino acid 720–769 of Human TRPV4 isoform 2 (NP_671737) was provided by Prof. Cheng-Kang Chiang (National Dong Hwa University, Hualien, Taiwan). The TRPV4 antibody was diluted in blocking buffer (1:100 dilution) and a 5-times excess of blocking peptide by weight was added to the antibody solution. The mixture was incubated with agitation overnight at 4 °C. The staining procedure was done with neutralized or unblocked antibody on the two samples.

### Immunohistochemistry

IHC was performed using the UltraVision Quanto Detection System HRP DAB (ThermoScientific, Cheshire, UK). Slides were first treated with hydrogen peroxide block reagent (ThermoScientific) and rinsed with PBS. Following that, Ultra V Block reagent (ThermoScientific) was used to block nonspecific binding. The slides were subsequently incubated with primary antibodies (the same as used in Western blotting), Primary Antibody Amplifier Quanto (ThermoScientific), and HRP Polymer Quanto (ThermoScientific) and DAB Quanto Chromogen and DAB Quanto Substrate (ThermoScientific) were used to visualize. The slides were then counterstained with hematoxylin.

### Statistical analysis

Data were expressed as mean ± standard deviation (SD). Because the sample distribution is not normal and the sample size is small, differences in protein expressions between mild KC, severe KC, and controls were analyzed using the nonparametric Mann–Whitney test. The association between the bladder protein expressions and urodynamic parameters was analyzed using the Pearson correlation. A P value of less than 0.05 was considered statistically significant.

## Supplementary Information

Supplementary Information.
