# Supplementary material for: Smaller bladder capacity and stronger bladder contractility in patients with ketamine cystitis are associated with elevated TRPV1 and TRPV4
Source: Sci Rep. 2021 Mar 4;11:5200. doi: 10.1038/s41598-021-84734-4 (PMC7933333; doi:10.1038/s41598-021-84734-4)
Supplement: Supplementary file 1 — Supplementary Information. [file 41598_2021_84734_MOESM1_ESM.docx]

**Supplementary Materials**

Title: Smaller Bladder Capacity and Stronger Bladder Contractility in Patients with Ketamine Cystitis Are Associated with Elevated TRPV1 and TRPV4

List of authors: Hsueh-Hui Yang, Jia-Fong Jhang, Yung-Hsiang Hsu, Yuan-Hong Jiang, Wei-Jun Zhai, Hann-Chorng Kuo*

Supplemental Figure S1. Western blot images from four controls, 12 mild KC, and 12 severe KC

Supplemental Figure S2. TRPV4 blocking peptide competition assay. Bladder sections from mild KC were stained with anti-TRPV4 antibody in the (A) absence or (B) presence of TRPV4 peptide.

Supplementary Table S1. Semi-quan quantification for immunofluorescence staining images from four controls, 12 mild KC, and 12 severe KC

Supplementary Figure S1. Western blot images from four controls, 12 mild KC, and 12 severe KC


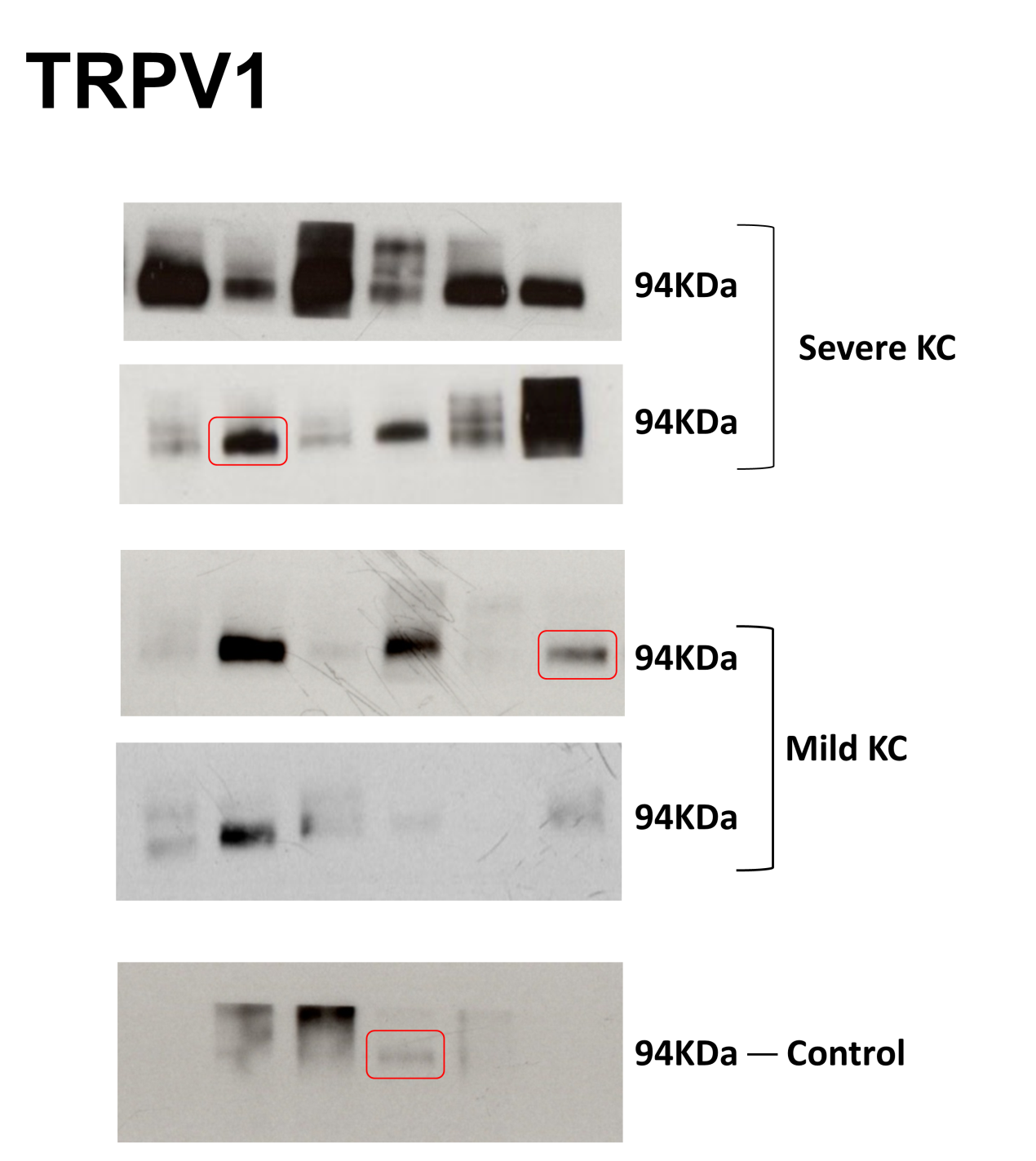


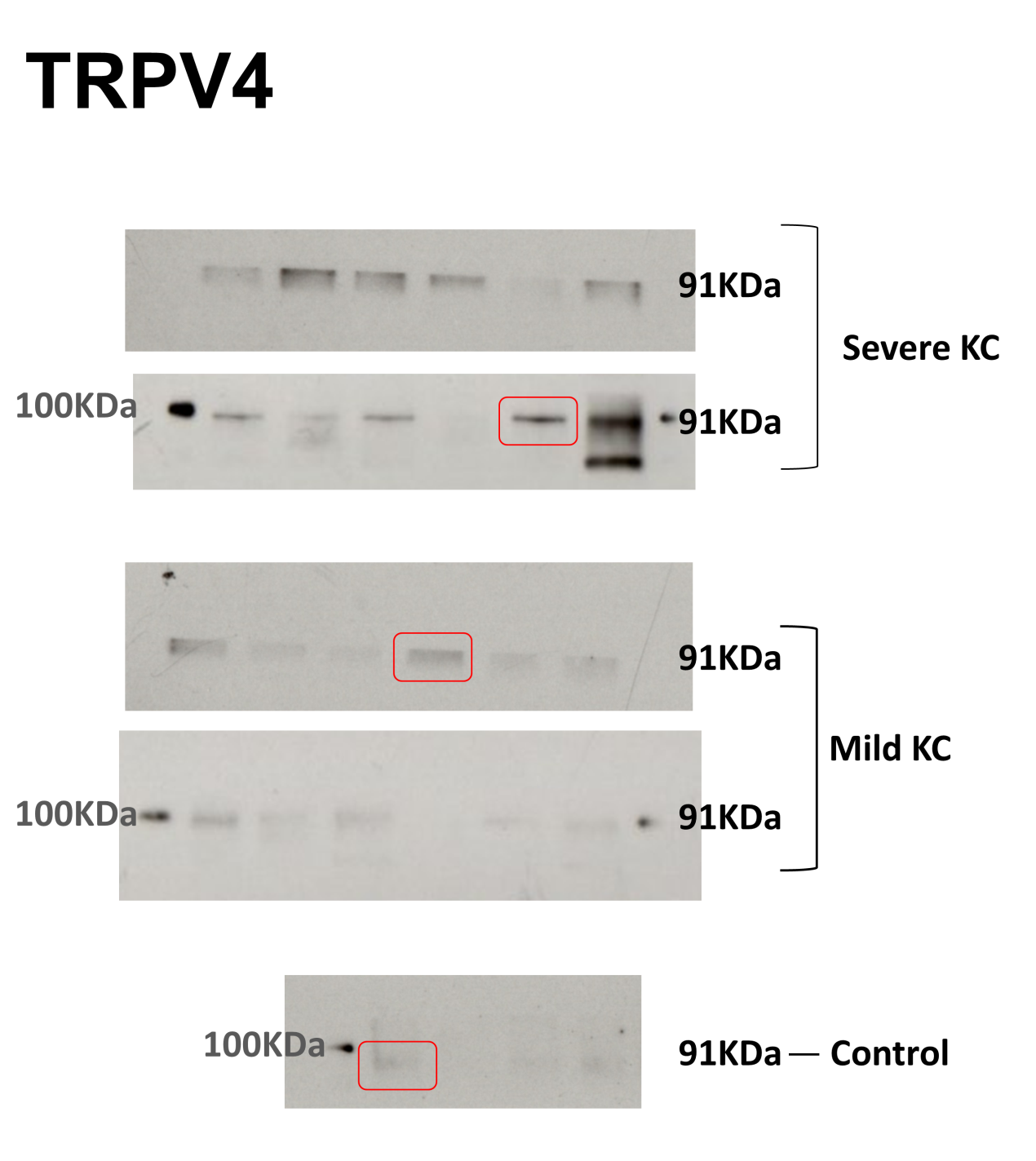

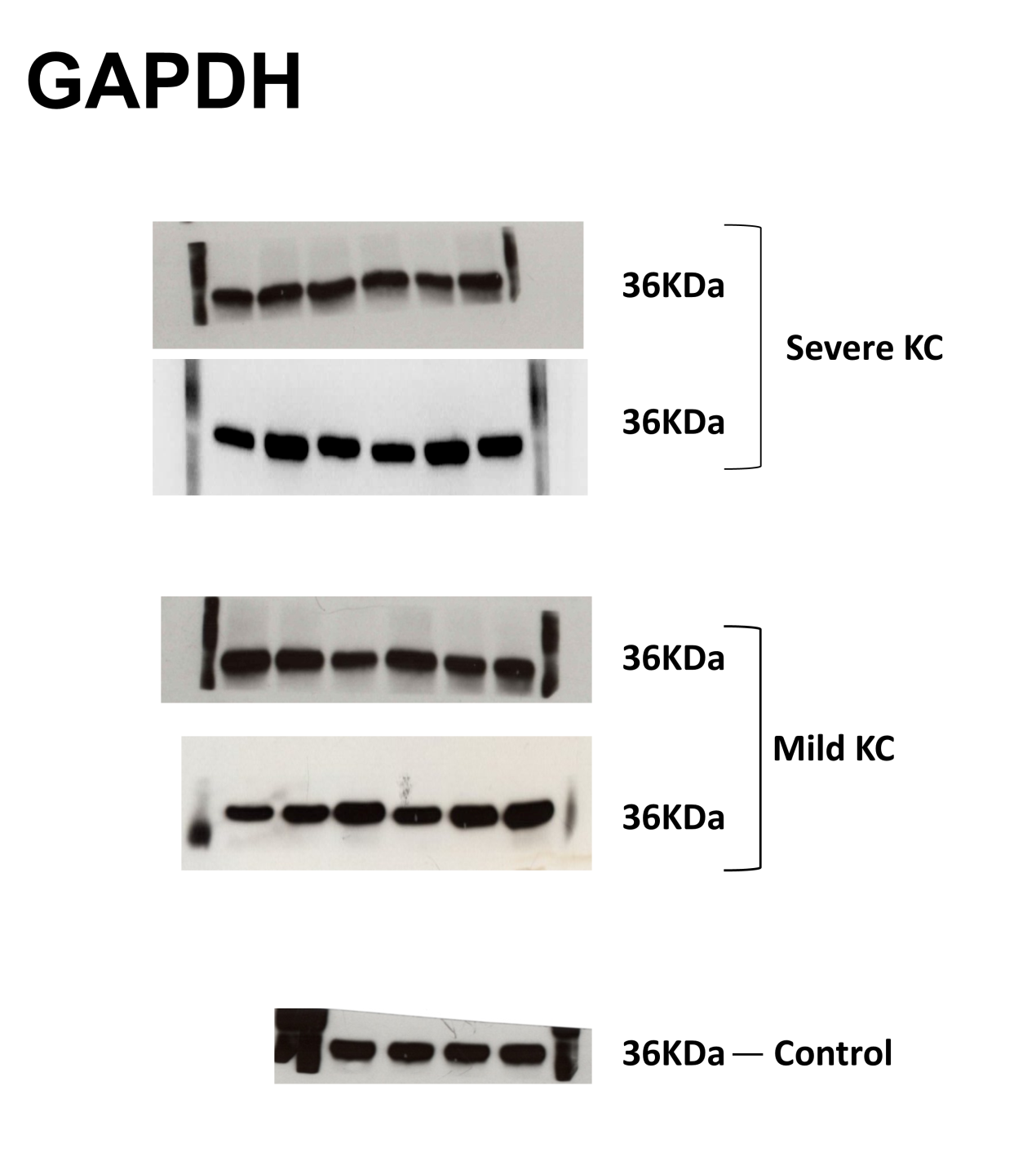


Supplementary Figure S2. TRPV4 blocking peptide competition assay. Bladder sections from mild KC were stained with anti-TRPV4 antibody in the (A) absence or (B) presence of TRPV4 peptide.


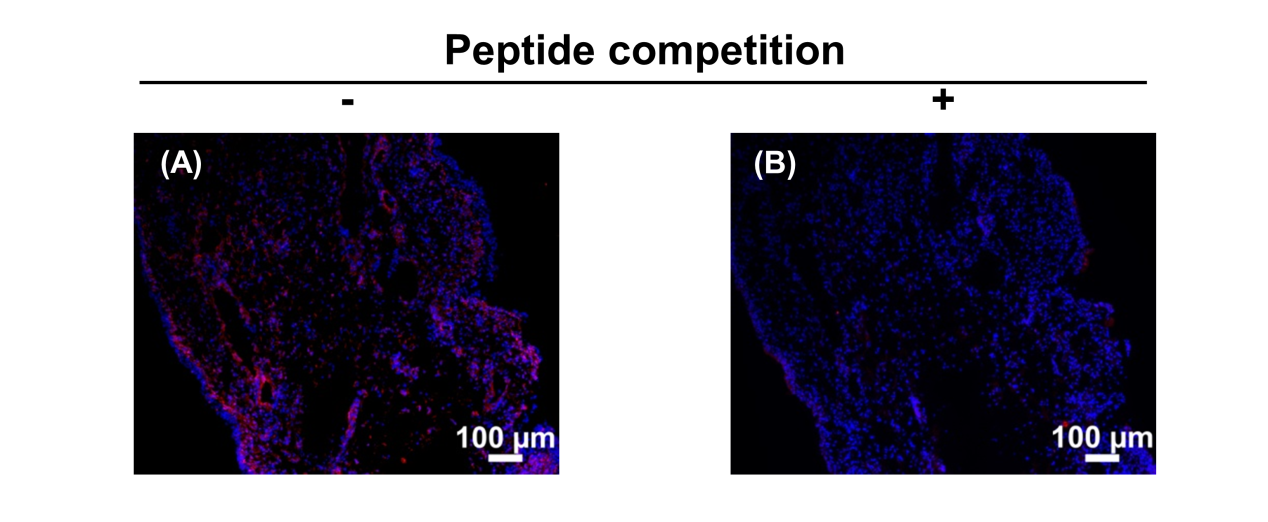


Supplementary Table S1. Semi-quan quantification for immunofluorescence staining images from four controls, 12 mild KC, and 12 severe KC

| Immunofluorescence staining |  | KC |  |
| --- | --- | --- | --- |
|  | Control | Mild KC | Severe KC |
| TRPV1 (fluorescence intensity/μm^2^) | 0.99 ± 0.20 | 1.43 ± 0.22 | 3.08 ± 0.87 |
| TRPV4 (fluorescence intensity/μm^2^) | 0.95 ± 0.19 | 1.35 ± 0.29 | 3.06 ± 0.80 |
